# Eating Disorders In weight-related Therapy (EDIT) Collaboration: Rationale and study design

**DOI:** 10.1017/S0954422423000045

**Published:** 2023-02-15

**Authors:** Natalie B. Lister, Louise A. Baur, Susan J. Paxton, Sarah P. Garnett, Amy L Ahern, Denise Wilfley, Sarah Maguire, Amanda Sainsbury, Katharine Steinbeck, Caroline Braet, Andrew Hill, Dasha Nicholls, Rebecca A. Jones, Genevieve Dammery, Alicia Grunseit, Kelly Cooper, Theodore K Kyle, Faith N. Heeren, Kylie E Hunter, Caitlin M McMaster, Brittany J Johnson, Anna Lene Seidler, Hiba Jebeile

**Affiliations:** 1 The University of Sydney, Children’s Hospital Westmead Clinical School, Westmead, Australia 2145; 2 Charles Perkins Centre, The University of Sydney, Sydney, Australia 2145; 3 Kids Research, Sydney Children’s Hospital Network, Westmead, Australia 2145; 4 School of Psychology and Public Health, La Trobe University, Melbourne, Victoria, Australia; 5 MRC Epidemiology Unit, University of Cambridge, Cambridge, CB2 0QQ, UK; 6 Washington University in St. Louis, St. Louis, USA; 7 InsideOut Institute for Eating Disorders, Boden Collaboration for Obesity, Nutrition and Eating Disorders, Charles Perkins Centre, The University of Sydney, Sydney, New South Wales, Australia; 8 The University of Western Australia, School of Human Sciences, Crawley, WA, Australia; 9 The Academic Department of Adolescent Medicine, The Children’s Hospital at Westmead, Westmead New South Wales Australia 2145; 10 Department of Developmental, Personality and Social Psychology, Ghent University, Henri Dunantlaan 2, 9000 Ghent, Belgium; 11 Leeds Institute of Health Sciences, University of Leeds, United Kingdom; 12 Division of Psychiatry, Imperial College London, 2^nd^ Floor, Commonwealth Building, Du Cane Road, London, W12 0NN, UK; 13 NIHR ARC Northwest London, UK; 14 The Children’s Hospital at Westmead, Nutrition and Dietetics, Weight Management Services, Westmead, NSW 2145, Australia; 15 Weight Issues Network, Australia; 16 ConscienHealth, Pittsburgh, Pennsylvania, USA; 17 Department of Health Outcomes and Biomedical Informatics, University of Florida College of Medicine, Gainesville, Florida, USA; 18 National Health and Medical Research Council Clinical Trials Centre, The University of Sydney, Sydney, New South Wales, Australia; 19 Caring Futures Institute, College of Nursing and Health Sciences, Flinders University, Adelaide, South Australia, Australia, 5042

**Keywords:** Eating disorders, obesity, individual participant data, meta-analysis

## Abstract

The cornerstone of obesity treatment is behavioural weight management, resulting in significant improvements in cardio-metabolic and psychosocial health. However, there is ongoing concern that dietary interventions used for weight management may precipitate the development of eating disorders. Systematic reviews demonstrate that, while for most participants medically supervised obesity treatment improves risk scores related to eating disorders, a subset of people who undergo obesity treatment may have poor outcomes for eating disorders. This review summarises the background and rationale for the formation of the Eating Disorders In weight-related Therapy (EDIT) Collaboration. The EDIT Collaboration will explore the complex risk factor interactions that precede changes to eating disorder risk following weight management. In this review, we also outline the program of work and design of studies for the EDIT Collaboration, including expected knowledge gains. The EDIT studies explore risk factors and the interactions between them using individual level data from international weight management trials. Combining all available data on eating disorder risk from weight management trials will allow sufficient sample size to interrogate our hypothesis: that individuals undertaking weight management interventions will vary in their eating disorder risk profile, based on personal characteristics and intervention strategies available to them. The collaboration includes the integration of health consumers in project development and translation. An important knowledge gain from this project is a comprehensive understanding of the impact of weight management interventions on eating disorder risk.

## Introduction

Obesity and eating disorders are both complicated by serious, short- and long-term health problems ^(1; 2; 3)^. The prevalence of both is increasing ^(4; 5)^, with some data suggesting rates of combined obesity and eating disorders are increasing faster than the prevalence of either obesity or eating disorders alone ^(6)^. For example, between 1995 to 2015 in a community sample of Australian adults, prevalence of obesity alone increased 1.7-fold, and binge eating episodes increased 3.5-fold, while prevalence of combined obesity and recurrent binge eating episodes increased 5.7-fold ^(7)^. Co-existence of obesity and a range of eating disorders is seldom acknowledged ^(8)^, with these conditions commonly stereotyped as existing on opposite ends of an eating disordered spectrum. A key distinction is that obesity is defined by a physical metric, whereas eating disorders are defined by well characterised cognitive and behavioural phenotypes ^(9)^. Obesity and eating disorders share many risk factors (e.g. weight concern, dieting) ^(8)^ and treatment approaches (e.g. health professional support, selfmonitoring, goal setting, and normalising eating patterns) ^(10)^. Of concern, the focus on weight loss during obesity treatment may lead to under-diagnosis of eating disorders in people with obesity ^(6)^. While only a small proportion of individuals may develop or have exacerbated eating disorder symptoms during weight management ^(11)^, the potential burden for this risk is high. Nevertheless, there is a paucity of research and limited treatment pathways for those affected by *both* obesity and eating disorders. Additionally eating disorders may develop over several years ^(12)^ and weight management interventions may only be a single experience in the eating disorder development pathway. However, this is a unique point of engagement with health services, where risk can be identified and addressed, thus representing an important research and practice gap.

The aim of this review is to provide the background, rationale and study designs for the Eating Disorders In weight-related Therapy (EDIT) Collaboration. We describe considerations for the nuances of eating disorder development, including risk factors from observational studies, and describe how such risk factors may be influenced by behavioural weight management. The EDIT Collaboration will bring together individual participant data (IPD) from relevant trials to understand how individual characteristics and components of weight management interventions may contribute to eating disorder risk. We hypothesise that individuals undertaking weight management interventions will vary in their eating disorder risk profile, based on personal characteristics and intervention strategies available to them. Further, we propose individual characteristics can be identified and intervention strategies can be adapted to reduce eating disorder risk.

## State of the literature: Obesity, weight management and eating disorders

### Eating disorder prevalence and complications in people with obesity

Worldwide, in 2016, 39% of men and 40% of women were affected by overweight, while 11% of men and 15% of women were affected by obesity ^(13)^; and prevalence is predicted to rise by the year 2030 ^(14)^. For children and adolescents (5-19 years), the prevalence of obesity was 5.6% in girls and 7.8% for boys ^(4)^. Complications of obesity include type 2 diabetes, non-alcoholic fatty liver disease, cardiovascular disease, sleep apnoea and depression ^(15; 16)^. There is growing evidence that prevalence of obesity is higher in populations with culturally diverse and lower socioeconomic backgrounds ^(13; 17)^.

Eating disorders include anorexia nervosa, atypical anorexia nervosa, bulimia nervosa, binge eating disorder, and several other categories of feeding and eating disorders ^(18)^. Eating disorders are severe mental and physical health conditions with a long duration ^(19)^ and high morbidity ^(2; 20; 21)^. Despite misconceptions that eating disorders are diseases of individuals with lower body weight, the prevalence of eating disorders is higher in both men and women with obesity compared to their healthy weight peers ^(22)^. In a survey of 12,337 adults in the US, the lifetime prevalence, i.e., proportion of people that had any eating disorder at any point in their life, was 2.2% in men and 4.9% in women ^(22)^. However, men and women with obesity had a higher prevalence of eating disorders compared to the general population, at 3.8% and 7.6%, respectively ^(22)^. Similarly in adolescents, the prevalence of eating disorders is associated with higher body mass index (BMI). For example, a study of 3,043 Canadian adolescents found 9.3% of male adolescents and 20.2% of female adolescents with obesity had a sub- or full- threshold eating disorder compared to respectively 2.1% and 8.4% of adolescents with a BMI in the normal range ^(23)^. Data from 5,191 Australian adolescents show those with overweight or obesity were more likely to experience an eating disorder ^(24)^. Further, several studies have identified an increase in disordered eating behaviours over time in community samples ^(7; 25)^, particularly among individuals with overweight and obesity. It has been reported that approximately one in four adolescents with obesity engages in binge eating behaviours or experiences loss of control with eating ^(26; 27)^. Binge eating and a loss of control with eating are also associated with weight gain and symptoms of the metabolic syndrome and are important drivers of continuing weight gain ^(2; 28)^. Moreover, emerging evidence suggests sociocultural factors such as food insecurity and childhood adversity influence both eating disorders ^(29; 30)^ and obesity ^(31; 32)^, and both eating disorder symptoms and weight gain have been exacerbated by the COVID-19 pandemic lockdowns ^(33 34; 35)^.

Compared to the general population, individuals with an eating disorder have an elevated risk of premature mortality, with a German study showing that the highest mortality risk (standardised mortality ratio) associated with anorexia nervosa was 5.35, compared to bulimia nervosa (1.49) and binge eating disorder (1.50) ^(36)^. Indeed, complications of anorexia nervosa are equally severe for individuals with weight within or above the normal range (i.e. atypical anorexia nervosa compared to anorexia nervosa) ^(37)^. People with binge eating disorder experience a high prevalence of both psychiatric comorbidities (e.g. mood, anxiety and substance use disorders) ^(38; 39)^ and physical comorbidities (e.g. type 2 diabetes, hypertension and chronic pain) ^(40)^. These comorbidities are also associated with obesity ^(41; 42)^, however higher weight only partly explains the association with binge eating disorder ^(42)^. Almost 30% of adults with binge eating disorder also report a history of childhood obesity ^(43)^. Thus, identifying eating disorders and reducing risk during weight management has potential to reduce or prevent a range of physical and psychological complications.

While there may be a growing recognition that people with obesity are at increased risk of developing eating disorders ^(6; 44)^, there has been limited progress in the identification, prevention and treatment of eating disorders in the context of weight management.

### Behavioural weight management

Multicomponent behavioural interventions are first-line treatment for adolescents and adults affected by obesity ^(45; 46; 47)^. These interventions typically recommend a combination of diet physical activity and behavioural modifications. A 2018 systematic review of adult behavioural weight management interventions of at least 12 months in duration showed that they were likely to produce significantly more weight loss compared to standard care (mean difference in weight change [MD], –2.39 kg [95% CI –2.86 to –1.93]; 67 studies; n=22,065). Eligible studies included participants recruited from primary care or a health care system, and intervention groups experienced less weight regain during the follow-up periods (beyond 12-18 months, pooled MD compared to control, –1.59 kg [95% CI, –2.38 to –0.79]; n=1408) ^(48)^. Further, the risk of developing diabetes over 1 to 9 years was substantially reduced (pooled risk ratio, 0.67 [95% CI, 0.51 to 0.89]; 9 trials; n=3140). Clinical practice guidelines for adolescents with overweight or obesity recommend a family-based approach to multicomponent behavioural interventions that address dietary, sedentary and sleep behaviours ^(47)^. A 2017 Cochrane review ^(49)^ found multicomponent behavioural interventions for adolescents (aged 12-17 years) with overweight or obesity resulted in a mean change in body weight of -3.67 kg (95% CI –5.21 to –2.13; 20 trials; n=1993) and BMI of -1.18 kg/m^2^ (95% CI –1.67 to –0.69; 28 trials; n=2774). These effects were maintained at 24-months follow-up. A 2012 systematic review reported significant improvements in low-density lipoprotein cholesterol (–0.30 mmol/L, 95% CI –0.45 to –0.15), triglycerides (–0.15 mmol/L, 95% CI –0.24 to –0.07), fasting insulin (–55.1 pmol/L, 95% CI –71.2 to –39.1) and blood pressure up to one year from baseline following lifestyle interventions for children and adolescents. Hence, multicomponent intensive behavioural weight management interventions can effectively reduce body weight and cardiometabolic risk in both adolescents and adults.

An emerging area of research and practice are dietary interventions with the potential to induce greater weight loss and improve cardiometabolic complications of obesity ^(50; 51; 52)^. These interventions may include very low energy diets (<800 kcal/d), very low carbohydrate diets (<50 g carbohydrate/d) or intermittent energy restriction; and require both medical and dietetic supervision ^(53; 54; 55)^. Such interventions are recommended for adolescents and adults with obesity and associated complications, or with severe obesity ^(47; 56)^. These restrictive approaches play an important role in effectively managing weight and cardiometabolic risk, particularly as an obesity management approach prior to bariatric surgery or when pharmacological and surgical approaches are not available or contraindicated. However, the effect of these interventions on eating disorder risk is unclear.

### Weight management and eating disorder risk

There is concern that dietary interventions, the cornerstone of behavioural weight management, may promote disordered eating and worsen psychological health ^(57; 58)^ in some individuals. This is informed by longitudinal data showing that dieting is an important step within eating disorder development ^(59; 60)^. However, some of these studies have poorly characterised the population sampled and the definition of “dieting”. Nevertheless, energy restriction may trigger binge eating in some people and it is thought that dietary and weight monitoring may trigger a preoccupation with food, weight and shape. Data from intervention studies including dietary components are described below.

#### Evidence from systematic reviews

Systematic reviews have examined the association between behavioural weight management and the change in eating disorder risk. A 2017 systematic review examining weight management interventions for adults identified five randomised controlled trials (RCTs), all of which reported beneficial outcomes for eating disorder symptoms, including a reduction in binge eating ^(61)^. Similarly, systematic reviews of pre-post studies and RCTs conducted in children and adolescents found no change or a small reduction in eating disorder symptoms, including binge eating and loss of control, following behavioural weight management ^(11; 62; 63; 64)^. Other related eating disorder risk factors, including depression, anxiety, and low body image and self-esteem have also been improved following weight management in both adults and adolescents ^(65; 66; 67; 68; 69)^. The effect of restrictive dietary approaches, including low or very low energy diets, on binge eating has been examined in a systematic review of 10 studies including 805 adults ^(58)^. In participants with pre-treatment binge eating disorder, studies reported a reduction in binge eating. The evidence was mixed in studies with participants with sub-clinical or no binge eating symptoms prior to treatment, with some studies showing a reduction in symptoms and others showing no change or an increase. Importantly, two studies included in this review reported an increase in binge eating or the onset of binge eating disorder in 10 to 15% of participants ^(58)^. To our knowledge, the evidence of restrictive dietary approaches on eating disorder risk in adolescents is yet to be synthesised. These interventions are likely to include delivery features very different from behavioural weight management interventions included in the reviews in adults above, and the implications of this are unknown.

In summary, previous evidence suggests that eating disorder risk is reduced for most participants following professionally supervised behavioural weight management; however, individual studies have reported a small subset of participants who develop an eating disorder during the intervention or in the years following intervention^(70; 71; 72; 73; 74; 75)^. For example, scores increased to above a clinical cut-point in seven children in one study and three of 56 participants followed up at 6-years had developed binge eating disorder in another study ^(71; 72)^. The mechanisms by which behavioural weight management may increase or decrease eating disorder risk at the individual level are not clear.

### Individual variation in responses to weight management interventions

Few studies have investigated individuals’ characteristics for associations with eating disorder outcomes following behavioural weight management interventions. For some individuals within the general population, dietary restraint (a proxy marker of dieting behaviours) is associated with the development of symptoms of binge eating disorder and bulimia nervosa ^(76; 77)^. In contrast, in others it may be an important behaviour that enables improvements in weight management and cardiometabolic health ^(78)^. This divergence in response may be in part explained by the difficulty in distinguishing between flexible (i.e. gradual reduction, foods are limited in quantity rather than eliminated) and rigid restraint (extreme, all or nothing mentality) ^(78)^. Psychosocial predictors thought to play a role in the development of eating disorders include poor self-esteem, depression, anxiety, bulimic behaviours (i.e. binge eating with compensatory behaviours), body dissatisfaction, and drive for thinness ^(60; 79)^. It is possible that the interactions or clustering of such individual characteristics with dietary restraint may be important for eating disorder development. For example, Stice’s Dual Pathway model hypothesises that pressure for thinness increases risk for body dissatisfaction, which in turn increases the risk for dietary restriction and/or negative affect, thereby increasing the risk for subsequent onset of binge eating-related disorders ^(80)^. However, etiological models including these risk factors do not consistently predict onset of eating disorders ^(12)^, suggesting varied individual responses. Importantly, research identifying eating disorder risk factors has been conducted in predominantly healthy weight populations; risk factors specific to individuals with obesity, for the full spectrum of eating disorder diagnoses are needed ^(44; 81; 82)^.

Beyond individual characteristics, there may be components of behavioural weight management interventions that influence eating disorder risk. Behavioural weight management interventions typically include a combination of intervention strategies (e.g., related to diet, movement, eating behaviour), delivered through various approaches (e.g., in terms of mode of delivery, session frequency/duration). Some commonly used strategies within weight management interventions are considered disordered behaviours in the context of eating disorder development, or may be components of etiological models of eating disorders. Two examples are the restriction of energy intake and self-monitoring of weight ^(83)^. In behavioural weight management interventions, the prescription of restriction of energy intake (i.e., reduced food intake), is thought to equate to dietary restriction, while a focus on monitoring of weight, is likened to pressure for thinness and weight preoccupation. Hence these two components of behavioural weight management are thought to tie into the Dual Pathway sequence to promote disordered eating behaviours ^(44)^. This is a contentious theory, with intervention studies of restricted energy intake for up to two years in individuals without obesity (such as the CALERIE trials ^(84; 85)^), demonstrating increases in dietary restraint without increases in binge eating or eating disorders ^(86)^. Indeed, this leads to the conceptualisation of dietary restraint as a successful self regulation strategy. Schaumburg et al ^(78)^ posit that when this self regulation fails, and is followed by a period of disinhibition, eating disorder risk is increased ^(78)^.

Moreover, intervention components such as self-monitoring improve weight-related outcomes and long term weight maintenance for most people ^(87)^. Large scale behavioural programs, such as the Diabetes Prevention Program (DPP) and Look AHEAD, demonstrate that self-monitoring of weight and dietary intake are positively related to weight loss ^(88; 89; 90)^, however these factors are also associated with disordered eating in community samples ^(83)^. Furthermore, factors such as increased self-esteem and self-efficacy around healthy eating are thought to be protective against disordered eating ^(91)^, suggesting that changes in individual characteristics may mediate changes in eating disorder risk during interventions. Indeed, eating disorder treatment programs may also include components of regular weighing and the development of healthy eating behaviours. This highlights the need to understand the complexities of interevention components of weight management interventions for eating disorder risk. There are also many components of behavioural weight management interventions which may be protective against disordered eating, including regular contact and support from healthcare professionals, and strategies such as promotion of self-efficacy, realistic goal-setting, supported problem-solving, and strategies for stimulus control or social support ^(92)^. Thus, a complex interaction between individual characteristics and intervention components is likely to influence eating disorder risk during weight management. To inform models of care for clinical practice it is vital to identify and understand components that may increase or decrease eating disorder risk for different individuals who participate in professionally supervised behavioural weight management programs. Addressing this is the central goal of the EDIT Collaboration.

## Stakeholder engagement in obesity and eating disorder research

To identify individual or intervention-level attributes that influence eating disorder risk in the context of weight management, it is essential to bring together stakeholders including those with lived experience of obesity and eating disorders. Engagement of consumers in research development and dissemination is an important process for evidence-based medicine ^(93; 94; 95)^.

The engagement of consumers in obesity and eating disorder research must be sensitive to the stigma and potential harms for those affected by both conditions. Data on the lived experience of people with combined obesity and eating disorders are limited. However, qualitative studies in people with lived experience of obesity frequently identify the harm caused by obesity stigma and weight bias ^(96; 97)^. A 2017 systematic review of cross-sectional studies reported that more frequent weight stigma experiences were associated with poorer physiological and psychological health ^(98)^. Indeed, using the term obesity is an important consideration for researchers, with studies reporting mixed responses to the term from people with higher weights ^(99; 100)^. Many people with obesity prefer neutral terms, such as ‘weight’ be used in clinical care. However one study found all weight related terms elicit negative emotions ^(99)^. On the other hand, obesity is a defined medical condition by a number of international health professional organisations, including the World Health Organization ^(101)^. Systematic reviews and international guidelines make suggestions and recommendations for reducing weight stigma and bias, such as the Joint international consensus statement for ending stigma of obesity ^(102; 103; 104; 105)^. The EDIT Collaboration will use person-first language for scientific discourse and language used within the collaboration will be reviewed and adjusted as required.

## Summary and rationale for the EDIT Collaboration

Behavioural weight management interventions form the first-line treatment approach for obesity ^(45)^. Clinical trials and systematic reviews show that, in addition to improved weight and cardiometabolic health, adolescents and adults with obesity who participate in supervised weight management interventions overall have improved eating behaviours, and psychological outcomes ^(11; 67; 69; 106)^. However, there is ongoing concern that these interventions may promote disordered eating and worsen psychological health in some individuals ^(57)^. Indeed, some studies report worsening eating disorder outcomes in some individual participants ^(11)^. This suggests that those who have poorer outcomes following weight management interventions are not captured when studies report *aggregate* risk scores. Due to the required large sample sizes and need for individual-level analysis (rather than pooling summary scores), the important research questions of whether weight management increases or decreases eating disorder risk for an individual is difficult if not impossible to answer using a single trial, qualitative methods, or standard aggregate data meta-analyses. Further, weight management interventions are complex and often poorly described. Research investigating whether certain intervention types or components of interventions may either increase or decrease eating disorder risk at an individual level is needed. It is likely that complex interactions between individual characteristics and intervention components influence eating disorder risk responses during weight management. Interventional evidence that addresses clinically supervised behavioural weight management for people with obesity should be examined to address these concerns.

## Research program

**Figure 1:**
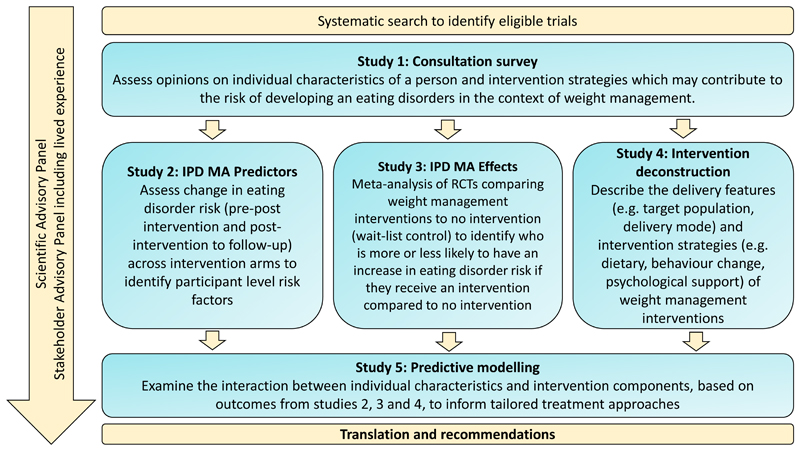
Research plan for the Eating Disorders In weight-related Therapy (EDIT) Collaboration. IPD MA, individual participant data meta-analysis; RCTs, Randomised control trials.

The EDIT Collaboration will bring together clinicians, researchers, biostatisticians and individuals with lived experience from around the world to improve treatment for people affected by obesity and eating disorders. The EDIT Collaboration aims to: (1) To understand which participants experience a change in eating disorder risk, or related symptoms, during and following weight management interventions ; (2) understand which intervention components may contribute to eating disorder risk; (3) identify predictive pathways for increased or decreased eating disorder risk during weight management; and (4) develop resources and recommendations to reduce eating disorder development during obesity treatment. To achieve these aims, the EDIT Collaboration will conduct five related studies (Figure 1). Detailed methodologies will be published separately but a brief overview is provided below.

### Scientific and stakeholder engagement

The work of the EDIT Collaboration is guided by Scientific and Stakeholder Advisory panels with international representation. The Scientific Advisory Panel includes experienced researchers and clinicians from the fields of obesity and eating disorders or those working across both conditions. The panel is responsible for overall program oversight and will provide strategic advice relating to the scientific rigour of each included study, contribute to protocol development, scientific publications, guide project output and the translation of project outcomes. The Stakeholder Advisory Panel comprises consumers with a lived experience of eating disorders, obesity or both conditions. The Stakeholder Advisory Panel will provide strategic advice, contribute to protocol development, and guide project outputs and translation from the viewpoint of the end consumers of health services.

### Eligible trials

Systematic searches of electronic databases and trial registries ^(107)^ are being conducted to identify trials that meet our inclusion criteria: **1**) randomised controlled trial of behavioural weight management intervention; **2**) for adolescents and/or adults with obesity; **3**) report at least one measure of eating disorder symptoms or behaviours at baseline and post-intervention or follow-up using a validated self-report questionnaire (e.g., Eating Disorder Examination Questionnaire, Binge Eating Scale) and/or clinical assessment or diagnostic interview (e.g., Eating Disorder Examination). The protocol for this review is registered with PROSPERO (CRD42021265340), accessible from https://www.crd.york.ac.uk/prospero/display_record.php?ID=CRD42021265340.

Representatives from each identified trial will be invited to join the collaboration and share IPD, i.e. line-by-line data for each individual participant data. The corresponding authors of identified trials are invited to join the EDIT Collaboration via email. If, after two attempts, no response has been received, other authors on the paper or listed on a registration record will be emailed. Finally, we will attempt to contact trialists via telephone, using our networks, via their institutions or at conferences. If trialists are unable to be contacted after multiple attempts, the trial will be excluded, since our analyses are not possible using published summary data alone. Trial representatives will have the opportunity to provide input to all major stages of the project including protocol development, analysis, results interpretation and translation.

### Study 1: Consultation

We will identify individual participant characteristics and intervention strategies which may contribute to an increase or decrease in eating disorder risk during weight management interventions through broad stakeholder consultation. Using an online survey, we will canvass diverse opinions on possible causes of eating disorder development in weight management interventions. Participants will be asked to rate the relevance of individual characteristics (e.g. body dissatisfaction, history of selfdirected dieting, disinhibition related to eating) and intervention strategies (e.g. dietary monitoring, dietary behaviour change strategies, informed by a psychological framework or theory) and identify any not listed in the survey. Individual characteristics and intervention strategies listed in the survey are informed by relevant literature, expert and consumer consultation (Scientific and Stakeholder Advisory Panels). The outcomes of this survey will be used to inform the analyses in the studies below.

### Studies 2 and 3: Individual participant data meta-analyses

Meta-analysis of IPD is considered the “gold standard” ^(108)^ for meta-analysis, in part due to the opportunity to explore differences in treatment effects across subgroups. These subgroups might include subsets of participants, such as those with higher eating disorder risk at baseline, or subsets of studies, such as those with particular intervention strategies ^(109)^.

We will collate all available data from all eligible studies to examine the individual risk of eating disorder development during weight management trials. IPD from collaborating trials will be collated into a central database. Trials will be identified through systematic searches, investigator networks and study branding (editcollaboration.com). The specific variables to be included in the analysis will be informed by stakeholder consultation (Study 1) and data availability, whereby any suggested predictor will be considered. We will conduct two IPD meta-analyses, with detailed methodology published elsewhere *a priori*. Study 2 aims to identify baseline participant risk factors which predict an increase or decrease in eating disorder risk, or related symptoms, during and following a weight management intervention. This will be a pre-post IPD meta-analysis. Study 3 aims to determine whether there are baseline participant risk factors which predict change in eating disorder risk, or related symptoms, if they receive any behavioural weight management intervention compared with no intervention (i.e., no treatment controls). This will be an IPD meta-analysis maintaining randomisation.

### Study 4: Intervention deconstruction

Interventions included in the EDIT Collaboration will be deconstructed into their discrete components to improve understanding of what they involve. Intervention components will include the intervention strategies to drive weight management (e.g. dietary or behaviour change strategies) and the features of how such interventions are delivered. Using a systematic coding framework developed for this study, we will then compare and synthesise the components of interventions targeting adolescent and adult populations. This project will allow for future quantitative analysis of intervention components and individual participant eating disorder risk.

### Study 5: Predictive modelling

Data from Studies 2, 3 and 4 will be combined to identify any interactions between individual characteristics and intervention strategies which may increase or decrease the risk of eating disorders during weight management. Detailed methodology informed by the findings of studies 1-4 will be published separately.

### Translation plan and recommendations

Knowledge gains (see **Box 1**) will inform the translation plan and recommendations. Workshops with the Stakeholder Advisory Panel (which includes stakeholders who are consumers with lived experience of obesity and eating disorders), Scientific Advisory Panel, and trial representatives will inform our translation action plan. A working group will be formed to *develop plans* for five key areas for translation: i) models of care for obesity management that consider eating disorder risk; ii) health professional education (e.g. training webinars, scientific publications, conferences, recommendations for screening and monitoring protocols); iii) community dissemination (e.g. website, newsletter, community seminars, decision aids for informing treatment consent); iv) identification of strategies to support further implementation; and v) policy briefing documents summarising key evidence that emerges from the research.

## Strengths and Limitations

The strengths of this research are first the use of robust statistical methods to quantitatively examine individual-level and intervention-level eating disorder risk during behavioural weight management interventions. We will use statistical methods recommended by the Cochrane Collaboration ^(108; 110)^, led by a team of biostatisticians with previous IPD meta-analyses experience ^(111; 112)^. Secondly, this project incorporates consumers’ views and broader stakeholder engagement to set research priorities and to translate the outcomes of the study. Thirdly, adolescents trials are included as an important life stage when the trajectories of both obesity and eating disorders become firmly established.

However, the studies outlined in this current research plan have limitations. This program will not report on qualitative experiences of participants who have undertaken weight management interventions. The IPD relies on retrospective analysis of data from clinical trials. Thus, there is an inherent risk that adverse eating disorder outcomes are not captured due to missing data, higher participant attrition among those at risk, or insufficient follow up of the included studies. However, IPD allow us to include data from excluded participants (e.g., outliers), more variables and timepoints from data sets that may not be included in a traditional aggregate data meta-analysis, thus increasing power to conduct subgroup analyses and detect adverse events. Further, where data are available, we will include in our analysis known psychosocial predictors of eating disorder development (e.g., self-esteem, depression, anxiety, bulimic behaviours, body dissatisfaction, and drive for thinness) to identify potential changes in an individual’s risk profile that may precede changes in global eating disorder risk. Future research addressing qualitative experiences and a prospective data analysis will be important to complement the current research plan.

There is also the possibility that the clinical trials /interventions eligible for the EDIT Collaboration (i.e. including a validated comprehensive measure eating disorder risk) are not representative of broader weight management interventions and findings will not be generalisable to all weight management. Moreover, the withdrawal of interventions and support may also influence eating disorder risk, and long-term data may not capture this changing risk profile. All included trials are providing a weight management intervention, thus, whether eating disorder risk would differ for those not referred or enrolled in an intervention will not be determined. Instead, this project will identify adolescents and adults presenting for weight management: i) for whom weight management will likely improve physical and mental health; ii) for whom behavioural weight management is not recommended, and; iii) whether intervention components can improve outcomes for different individuals.

## Summary and conclusion

The EDIT Collaboration will combine IPD meta-analysis and intervention coding to quantitatively explore the underlying pathways that increase or decrease eating disorder risk during behavioural weight management interventions. By understanding how individual participant characteristics may interact with intervention components to influence eating disorder risk, we have the potential to create an innovative toolbox for clinicians to build the safest interventions for each individual. Future combined research between obesity and eating disorder fields has the potential to lead to a tailored precision therapeutic response, improving both obesity and eating disorders care.

## Box 1 EDIT Collaboration knowledge gains

Expected knowledge gains

- Diverse community perspectives on the role of individual characteristics and intervention strategies that may increase or decrease eating disorder risk
- Comprehensive understanding of how weight management interventions impact eating disorder risk at an individual level
- Identify participant level predictors of change in eating disorder risk in the context of weight management
- Understand intervention strategies used during weight management interventions and how they may contribute to eating disorder risk
- Find interactions between individual characteristics and intervention components that contribute to an individuals increase or decrease in eating disorder risk
- Development of an international database of weight management interventions measuring eating disorder risk
- Development of recommendations for tailored models of care for obesity treatment
